# Influence of size, sex, and reproductive status on the thermal biology of endemic Florida scrub lizards

**DOI:** 10.1002/ece3.6897

**Published:** 2020-10-11

**Authors:** Alison M. Gainsbury

**Affiliations:** ^1^ Department of Integrative Biology University of South Florida St. Petersburg FL USA

**Keywords:** endemic lizard, gravid female, *Sceloporus woodi*, scrub habitat, thermoregulation, thermoregulatory precision

## Abstract

Climate change is impacting species globally, with many populations declining at an accelerated rate toward extinction. Ectothermic species are particularly vulnerable given their reproductive success is linked to environmental temperatures. Studies of the effect of temperature on reproductive success in oviparous squamates have focused mostly on nest temperatures, after eggs are deposited. However, in some species gravid females are known to thermoregulate differently than other adults to increase reproductive success. It is essential to understand what influences the thermal biology of breeding adults in a population to implement targeted conservation strategies. The Florida scrub lizard *Sceloporus woodi* is an endemic species listed as near‐threatened due to decreasing populations. This study is the first to document the thermal biology of these breeding adults in relation to size, sex, and reproductive status. A *t* test was used to determine whether sexual dimorphism was present in the sampled *S. woodi*. Full linear mixed‐effects models were used to test the influence of size, sex, and reproductive status on the thermal biology of *S. woodi*. Despite female‐biased sexual size dimorphism, there were no sex‐based differences in body temperature in the studied population. Interestingly, reproductive status influenced thermal biology of females during the breeding season, with gravid females maintaining lower body temperatures compared to nongravid females. However, gravid females did not regulate their body temperatures more precisely compared to nongravid females. These results indicate the population viability of this endemic species is potentially linked to the different thermoregulatory requirements of gravid females as compared to other adults. Lower body temperatures of gravid females, exacerbated by their lack of thermal precision, have disconcerting conservation implications in the face of climate warming. Future studies focusing on gravid females are warranted to attain effective biodiversity conservation strategies mitigating the impacts of climate warming.

## Graphical abstract

Interestingly, reproductive status was an important influence on thermal biology during breeding season, with gravid females maintaining lower body temperatures compared to nongravid females and males. Furthermore, gravid females did not regulate their body temperatures more precisely compared to nongravid females. Gravid females’ lower body temperatures exacerbated by their lack of thermal precision differences compared to nongravid females have disconcerting conservation implications for the endemic Florida scrub lizard in the face of climate warming.

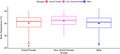

## 1 INTRODUCTION

Climate change is driving many species to extinction at an alarming rate. Thomas et al. (2004) estimated that 15%–37% of all species will be on an unavoidable decline toward extinction by 2050. Of these species, ectotherms are particularly vulnerable to climate change as they depend on the environment to regulate their body temperature to perform vital functions (Deutsch et al., 2008). It is crucial that ectotherms maintain their body temperatures at an optimal range within the variation of environmental temperatures for reproductive success (Andrews & Schwarzkopf, 2012; Hertz et al., 1993).

Reproductive success is also determined by the interplay between natural and sexual selection, which can lead to sexual dimorphism (Cox et al., 2003; Jiménez‐Arcos et al., 2017). When females are larger than males, fecundity selection is often invoked to relate the larger female sizes to more offspring (refer to Pincheira‐Donoso & Hunt, 2017). In ectothermic species, body size is known to influence thermal biology (Huey, 1982; Huey & Stevenson, 1979). Small‐bodied ectotherms warm up quickly for short periods of time when environmental temperatures are favorable and seek shelter when environmental temperatures are extreme (Stevenson, 1985). However, the combined influence of body size, sex, and reproductive status on the thermal biology of oviparous species is rarely examined, as most studies focus on viviparous species (Mathies & Andrews, 1997; Wang et al., 2014). In oviparous species, the effects of temperatures on reproductive success are mostly directed toward understanding nest temperatures, after the eggs are laid (Oufiero & Angilletta, 2010; Warner & Shine, 2009).

Nevertheless, there are oviparous lizard species where gravid females regulate their body temperatures differently than other adults in the population to benefit embryonic development as well as hatching success (refer to Angilletta et al., 2000; Rodríguez‐Díaz & Braña, 2011; Rodríguez‐Díaz et al., 2010; Telemeco et al., 2010). Of the few such species studied, some gravid females maintain higher body temperatures (Dayananda et al., 2017; Juri et al., 2018; Werner, 1990; Woolrich‐Piña et al., 2012), whereas other species maintain lower body temperatures (Schall, 1977; Smith & Ballinger, 1994; Smith et al., 1993). A selective advantage for maintaining higher body temperatures while gravid may be that it results in fewer incubation days (Shine, 1980). Alternatively, in regions with hot environmental temperatures during breeding season, lower body temperatures of gravid females may reduce thermal stress resulting in improved embryonic survival and offspring fitness (refer to Daut & Andrews, 1993). However, there are some species that show no differences in body temperature between gravid females and other adults (Andrews et al., 1999; Gillis, 1991; Lemos‐Espinal et al., 1997; Woolrich‐Piña et al., 2015). The ability of gravid females to thermoregulate precisely is another measure of thermal biology related to hatchling success in eggs prior to oviposition (Mathies & Andrews, 1997). In *Anolis nebulosus*, gravid females thermoregulated more efficiently despite a lack of differences in body temperatures compared to all adults (Woolrich‐Piña et al., 2015). This lack of pattern across taxa indicates the influence of sex on thermal biology may be species‐specific (refer to Schwarzkopf & Andrews, 2012). Thus, it is fundamental to gather more data to elucidate species thermal requirements to ensure reproductive success, particularly for species of conservation concern, in the face of climate warming.

There are many studies on the endemic Florida scrub lizard, *S. woodi*; nevertheless, no research has been conducted on how body size, sex, and reproductive status influence the thermal biology of this oviparous lizard. In addition, more information is needed on the ability of oviparous species to control their body temperatures when gravid. For ectotherms, where environmental temperature is directly related to population viability, the role that thermal biology plays to ensure reproductive success has large implications for biodiversity conservation (refer to Andrews & Schwarzkopf, 2012; Rodríguez‐Díaz & Braña, 2011). The aim of this study is to add to existing information by testing the influence of phenotype and reproductive status on the thermal biology of an oviparous species. I predicted that females and males would differ in body size, and body temperatures. Moreover, I expected gravid females body temperature would differ when compared to nongravid females and males, along with degree of thermoregulatory precision.

## 2 MATERIALS AND METHODS

### 2.1 Study species

The Florida scrub lizard, *S. woodi*, Stejneger, 1918 is endemic to the Florida scrub ecosystem. Females can lay multiple clutches from mid‐April to August with two to eight eggs per clutch, with a mean clutch size of four eggs (Jackson & Telford, 1974). *Sceloporus woodi* is classified on the International Union for Conservation of Nature Red List as near‐threatened (Hammerson, 2007). Their geographic range is restricted and disjunct across central and southern Florida (DeMarco, 1992a), and current population sizes are declining due to habitat loss (Bartlett & Bartlett, 1999; McCoy et al., 2004). Furthermore, the terrestrial (i.e., nonarboreal) niche of *S. woodi*, as compared to *S. jarrovi* and *S. undulatus*, provides an important ecological property to test patterns of thermoregulation.

### 2.2 Study site

The Florida scrub ecosystem is globally recognized as one of the most imperiled habitats (Ricketts et al., 1999). The original Florida scrub habitat is a series of marine deposit ridges, of which the largest is Lake Wales Ridge (Mushinsky & McCoy, 2017). This xeric, fire‐dependent, heterogeneous habitat (Myers, 1990) is severely impacted by human disturbance such as urban sprawl and agricultural development (Mushinsky & McCoy, 2017). The study site was located at Archbold Biological Station, Florida (27°10′50″N, 81°21′0″W), which has an average annual temperature (1952–2018) of 19.8°C and rainfall of 136.2 cm, with most rainfall occurring during June to September (https://www.archboldstation.org/html/research/monitoring/climate.html). Archbold Biological Station contains large areas of pristine scrub habitats from the original upland habitats of the Lake Wales Ridge (Abrahamson et al., 1984). *Sceloporus woodi* were captured and released in the scrubby flatwoods. This habitat burns regularly and is dominated by a combination of oaks, palmettos, and scattered Florida slash pine (Abrahamson et al., 1984).

### 2.3 Data collection

I captured adult lizards by hand or noose in June 2019 and released them at their site of capture in September 2019. Adults were classified by snout–vent lengths of 45 mm or larger (DeMarco, 1992a). I placed lizards in plastic terraria (36 × 22 × 24 cm) with a maximum of two individuals each and transported them to the laboratory at the University of South Florida St. Petersburg. In the laboratory, 36 glass terraria (56 × 30 × 34 cm) housed a single female in each terrarium, with some females joined by a single male. The terraria included 4–5 cm of sand substrate, water dish, and plastic cover object for basking and refuge. Illumination was provided by broad‐spectrum fluorescent light bulbs (Zoo Med ReptiSun 10.0 UVB Bulb) resting on top of the terraria and one heat lamp (75 W) suspended over the end of each terrarium enabling thermoregulation. Heat lamps maintained temperature gradients within terraria averaging a minimum of 30°C and maximum of 40°C during the daytime portion of a 14/10‐hr day/night cycle. I fed lizards daily crickets dusted with vitamin powder and provided water ad libitum. I measured lizard size by recording their snout–vent length (SVL) with a ruler to the nearest 0.1 mm the day after arrival to the laboratory (June) and the day before release (September).

I recorded lizard body temperature from 19 females and 18 males on alternate days during the breeding season in the morning and afternoon, from 10 June to 13 September 2019. Demarco (1992b) documented that *S. woodi* had an average of 11.7 days from ovulation to oviposition. Thus, gravid female body temperatures were averaged over the 11 days immediately preceding oviposition (and likewise for postgravid females for reasons of symmetry). To minimize handling stress on lizards, I measured all body temperatures using an infrared (IR) thermometer (Raytek Raynger ST61) pointed at the center of the dorsal abdomen (Hare et al., 2007).

### 2.4 Statistical analyses

I used a *t* test to determine whether sexual dimorphism was present in the sampled *S. woodi*. To test the influence of size, sex, and reproductive status on the thermal biology of *S. woodi*, I used full linear mixed‐effects models on body temperature and thermoregulatory precision (as response variables) with size, sex, and reproductive status as fixed effects and individual ID as a random effect. Parameters were log‐transformed and the model was checked with variance inflation factors (vifs < 3) and diagnostic plots for residual normality, and heteroscedasticity. I used an ANOVA type II to determine the statistical significance from the models. However, for clarity I report the untransformed parameters in the results. Body temperature variances were used to calculate thermoregulatory precision (Hertz et al., 1993). Means ± *SE* are reported in the results. All statistical tests were conducted using the significance level of 5% and performed in R 3.6.3 (R Development Core Team, 2020).

## 3 RESULTS

**FIGURE 1:**
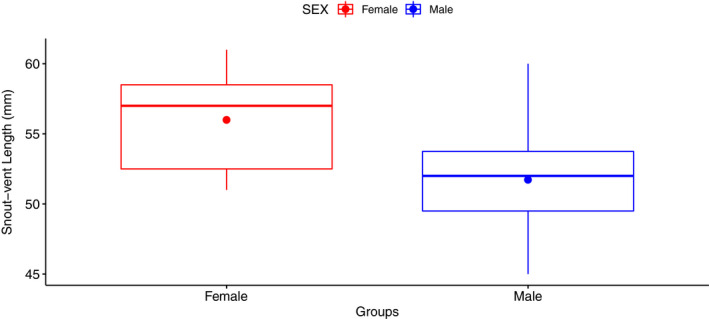
Body size (snout–vent length; mm) for female and male *Sceloporus woodi*. Sample sizes: females, *n* = 19; males, *n* = 18, with stars representing the means

Female *S. woodi* were larger than males in SVL (females: 56.00 mm ± 0.78; males: 51.72 mm ± 0.91; *t* = 3.56, *p* = .001; Figure 1). Even though sexual size dimorphism does occur in *S. woodi*, there was no effect on body temperature (Table 1). Also, when comparing all females (gravid and nongravid) to males, there were no differences in body temperature between sexes (Table 1).

**TABLE 1:** Parameters of fixed effects in linear mixed effect model of *Sceloporus woodi* body temperatures as response variable with sex and reproductive status as fixed effects and individual ID as a random effect. P represents statistical significance determined by an ANOVA type II

Model parameter	Estimate	*SE*	*t* value	*p*
Intercept	1.310	0.192	6.838	
Sex: M	−0.007	0.008	−0.851	.395
Reproductive status: nongravid	0.012	0.004	2.888	.004
Snout–vent length	0.133	0.110	1.211	.226

**FIGURE 2:**
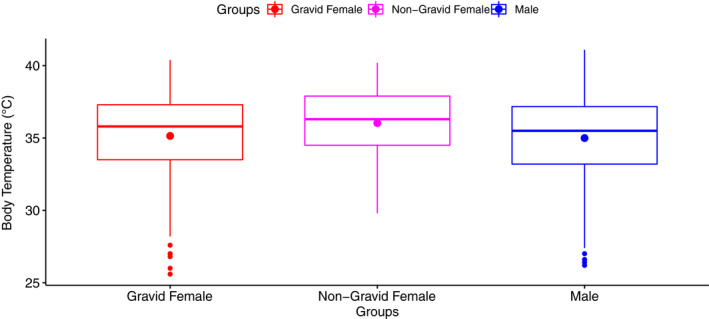
Body temperature (°C) for gravid, nongravid females and males *Sceloporus woodi*. Sample sizes: females, *n* = 19; males, *n* = 18 with stars representing the means

Interestingly, there was a strong effect of reproductive status on body temperature with gravid females (35.14°C ± 0.28) and males (35.00°C ± 0.12) recording lower body temperatures compared to nongravid females (36.03°C ± 0.21; Figure 2). However, neither gravid females compared to nongravid females nor males regulated their mean body temperatures variance more precisely, given the lack of difference in mean body temperatures variances (Table 2). Gravid female body temperature variance was 6.37°C ± 1.43 compared to nongravid females with a 4.69°C ± 0.50.

**TABLE 2:** Parameters of fixed effects in linear mixed effect model of *Sceloporus woodi* thermoregulatory precision (body temperature variances) as response variable with sex and reproductive status as fixed effects and individual ID as a random effect. P represents statistical significance determined by an ANOVA type II

Model parameter	Estimate	*SE*	*t* value	*p*
Intercept	1.789	2.024	0.884	
Sex: M	−0.089	0.088	1.009	.313
Reproductive status: nongravid	−0.056	0.080	−0.693	.488
Snout–vent length	−0.639	1.158	−0.552	.581

## 4 DISCUSSION

*Sceloporus woodi* is an oviparous lizard species listed as near‐threatened, with populations that are decreasing in size due primarily to habitat loss (Hammerson, 2007; McCoy et al., 2004). This endemic species inhabits the imperiled Florida scrub ecosystem generating a restricted and disjunct geographic distribution (DeMarco, 1992a). This study documents, for the first time, their thermal biology in relation to size, sex, and reproductive status. In the studied population, there were no differences in body temperature between sexes, despite female‐biased sexual size dimorphism. Sex was an important determinant of thermal biology when reproductive status is integrated, with both gravid females and males maintaining lower body temperatures compared to nongravid females during the breeding season.

Sex and reproductive status in *Sceloporus* have previously been reported to affect individual thermoregulatory behavior. In some species, gravid *Sceloporus* females exhibited higher body temperatures compared to nongravid females and males (*S. gadoviae*: Woolrich‐Piña et al., 2012; *S. jarrovii:* Beal et al., 2014). Alternatively, in other species gravid *Sceloporus* females were documented to maintain lower body temperatures compared to other adults in the population (*S. scalaris*: Smith et al., 1993; *S. virgatus*: Smith & Ballinger, 1994). These researchers did not compare the same female while gravid and nongravid, instead, made comparisons between different females. Interestingly, when comparing the same female while gravid and nongravid, *S*. *woodi* selected lower mean body temperatures when gravid, even though higher body temperatures would have been easily attainable in the terraria. Some females shared terrarium space with males, which could have excluded the females from warmer areas. The data suggest this did not occur, since males also had lower body temperatures when compared to nongravid females. Future investigation is needed on field temperatures to test whether gravid females maintain lower body temperatures during the breeding season (refer to Hertz, 1992; Hertz et al., 1993).

Furthermore, there are several nonmutually exclusive mechanistic hypotheses for what is driving lower mean body temperatures in gravid females as compared to nongravid females. For one, developing eggs in utero may be more sensitive to sudden increases in temperatures. If so, there should be a selective advantage for females to lower their body temperature to reduce the risk of thermal stress on embryonic development (Schall, 1977). There also exists the possibility that embryonic development might require lower temperatures to successfully start and/or maintain development before oviposition (Rafferty & Reina, 2012). The benefit does not necessarily need to be directed toward the offspring, but instead could be directed toward the gravid females (refer to Schwarzkopf & Andrews, 2012). Some gravid females were observed to eat less or stop eating before oviposition possibly due to avoid predation. Beal et al. (2014) demonstrated fasted *S. jarrovii* had lower preferred body temperatures when compared to fed individuals. However, Schuler et al. (2011) studied the same species and found no evidence of fasting altering body temperatures. It is likely that multiple processes exist concurrently driving gravid females of *S. woodi* to maintain lower body temperatures compared to nongravid females.

Thermoregulatory precision is another important attribute of thermal biology linked to reproductive success. Increased thermoregulatory precision by gravid females has been documented in reptile species, both in laboratory and field studies (Beuchat, 1986; Gier et al., 1989; Juri et al., 2018; Mathies & Andrews, 1997; Stewart, 1984; Woolrich‐Piña et al., 2015). Juri et al. (2018) studied *Tropidurus spinulosus*, an oviparous lizard species, and demonstrated reproductive females with larger body widths were able to thermoregulate more precisely, albeit this comparison was between different females. In this study, the same female is compared while gravid and nongravid with the result of no difference in thermoregulatory precision for the Florida scrub lizard. The lack of differences in thermal precision between gravid and nongravid females may be troublesome for this endemic species. However, two viviparous lizards have been previously documented to lack differences in thermal precision when gravid (*Eulamprus tympanum*: Schwarzkopf & Shine, 1991; *Chalcides ocellatus:* Daut & Andrews, 1993). Further investigation is needed to validate if *S. woodi* lacks the ability, in the field, to thermoregulate precisely when gravid; and if so, to determine what the consequences might be to hatching success and fitness.

In summation, lower body temperatures of gravid female *S. woodi* along with their lack of thermal precision differences compared to nongravid females may lead to detrimental effects on the population viability of this near‐threatened endemic species, in the face of future climate warming. As of 2015, global atmospheric temperatures rose 1°C above pre‐industrial levels. There is an expected increase ranging from 0.3°C to 4.7°C, with a likely increase of at least 1.5°C by 2100 (IPCC, 2018). The impacts of climate warming on embryos in utero will likely exacerbate the thermal challenges faced by gravid females of this oviparous species. Sinervo et al. (2010) stated climate warming that forced gravid females to be active at high body temperatures would lead to reductions in hatching success. Furthermore, the offspring that manage to hatch are predicted to be of low fitness quality. A study with a congeneric species, *Sceloporus undulatus*, found reduced embryonic survival and hatchling size under recurrent sublethal warming of nests, estimating a 24% decrease in annual survivorship (Carlo et al., 2018). Many tropical species are thermoregulating close to their maximum temperatures within their optimal ranges; thus, even trivial changes in thermoregulation to mitigate the impacts of climate warming on offspring may not be possible (Huey et al., 2009). This uncertain future has already caught up to some populations of cool‐adapted lizards on isolated mountaintops (Sinervo et al., 2010). The endemic Florida scrub lizard, *S. woodi*, is a subtropical near‐threatened species due to fragmented and restricted distribution ranges. Climate warming impacts on gravid females has the potential to further decrease their population sizes. It is fundamental that research integrates the influence of reproductive status on thermal biology of endemic species, concurrently ensuring reproductive success and biodiversity conservation.

## CONFLICT OF INTEREST

The author declares no conflicts of interest.

## AUTHOR CONTRIBUTION

**Alison Gainsbury:** Conceptualization (lead); Data curation (lead); Formal analysis (lead); Funding acquisition (lead); Investigation (lead); Methodology (lead); Project administration (lead); Resources (lead); Validation (lead); Visualization (lead); Writing‐original draft (lead); Writing‐review & editing (lead).

## Data Availability

Data are available from the Dryad Digital Repository at https://doi.org/10.5061/dryad.dbrv15f00
